# Profiles of Time Management Dispositions and Influencing Factors Among Nursing Interns: A Latent Profile Analysis

**DOI:** 10.1155/jonm/5020832

**Published:** 2026-11-04

**Authors:** Miaomiao Xiang, Luqing Chen, Xiaoxiao Xia, Le Xu, Mengru Wu, Peipei Ding, Ziqiao Sun

**Affiliations:** ^1^ Department of Neurology The First Affiliated Hospital of Anhui Medical University Hefei Anhui China ahmu.edu.cn; ^2^ School of Nursing Anhui Medical University Hefei Anhui China ahmu.edu.cn; ^3^ Department of Nursing The First Affiliated Hospital of Anhui Medical University Hefei Anhui China ahmu.edu.cn; ^4^ Department of Nursing The First Affiliated Hospital of University of Science and Technology of China Hefei Anhui China ustc.edu.cn; ^5^ Department of Burns The First Affiliated Hospital of Anhui Medical University Hefei Anhui China ahmu.edu.cn; ^6^ Department of Anesthesiology The First Affiliated Hospital of Anhui Medical University Hefei Anhui China ahmu.edu.cn

**Keywords:** influencing factors, latent profile, nursing interns, time management

## Abstract

**Background:**

In complex clinical settings, nursing interns often encounter difficulties with time management, leading to decreased efficiency, increased psychological distress, and impaired role transition. However, existing literature has largely treated this population as a homogeneous entity, overlooking the latent heterogeneity in time management dispositions and the specific influencing factors underlying these distinct profiles.

**Methods:**

This cross‐sectional survey was conducted from May to July 2025. We recruited 291 nursing interns from a tertiary hospital in Anhui Province, China. Latent profile analysis (LPA) was employed to identify distinct time management profiles. Subsequently, multivariate logistic regression was performed to examine factors associated with profile membership.

**Results:**

Three distinct profiles were identified: Passive Procrastination (16.5%), Guidance‐Dependent (60.1%), and Proactive Planning (23.4%). Using the Proactive Planning Profile as the reference group, multivariate logistic regression indicated that the Passive Procrastination profile was significantly associated with lower Family APGAR scores and self‐efficacy. Furthermore, the Guidance‐Dependent profile was associated with a lack of intention for further education, as well as lower self‐efficacy and Family APGAR scores.

**Conclusion:**

This study highlights the significant heterogeneity in the time management patterns of Chinese nursing interns. Specifically, the Proactive Planning profile was characterized by a strong intention for further education, high self‐efficacy, and higher family functioning. These findings provide an evidence‐based foundation for developing targeted interventions to address time management challenges in this population.

**Implications for Nursing Management:**

Nurse managers should replace one‐size‐fits‐all preceptorship with differentiated strategies that build interns’ self‐efficacy, engage families as a supplementary source of support, and provide behavioral guidance rather than motivational reinforcement for interns with procrastination tendencies. Longitudinal and interventional studies are warranted to validate these findings and refine such strategies.

## 1. Introduction

Time management is a core competency essential for patient safety and quality of care in clinical practice [1, 2]. In fast‐paced, high‐workload clinical settings, effective time management optimizes workflow and mitigates adverse events. It also serves as a key strategy for balancing professional and academic responsibilities while alleviating burnout [3–5]. For nursing interns transitioning from academia to practice, a period characterized by complex tasks and role adjustments, time management skills are particularly critical. However, evidence indicates that nursing interns frequently experience high levels of fatigue, predisposing them to medication errors [6]. These interns typically demonstrate suboptimal proficiency, experiencing work backlogs due to ineffective prioritization, which subsequently contributes to burnout [7] and turnover intention [8]. Therefore, investigating the characteristics and mechanisms of time management in this population is vital for improving clinical education quality and ensuring workforce stability.

Time management is defined as a self‐regulating process encompassing goal setting, planning, and monitoring [9, 10]. Previous research has identified antecedents across multiple dimensions. Regarding personality traits, conscientiousness is the most robust predictor of time management efficacy [11]. Psychologically, self‐regulation is considered the internal driver for optimizing time resource allocation [12]. Furthermore, women typically exhibit a slight advantage in time management [11]. Collectively, these findings provide a theoretical foundation for understanding the determinants of time management.

Despite these valuable insights, the current literature exhibits two primary limitations regarding research subjects and methodology. First, most existing studies focus on registered nurses [13, 14] or general student populations [15, 16], leaving empirical evidence regarding nursing interns scarce. Second, and more critically, prior research predominantly employs a variable‐centered analytical paradigm. This approach assumes population homogeneity, potentially masking individual heterogeneity. By neglecting distinct patterns across subgroups, traditional methods are limited in their ability to identify high‐risk individuals with low time management efficacy or derive insights from efficient subgroups. Consequently, this hinders the development of targeted interventions.

To address these limitations, a person‐centered approach utilizing latent profile analysis (LPA) [17] is essential to overcome the limitations of traditional variable‐centered paradigms. By analyzing individual response patterns across time management dimensions, LPA classifies the population into mutually exclusive profiles, thereby revealing intra‐group heterogeneity. Following profile identification, exploring the factors associated with these categories is crucial for developing targeted management strategies. Guided by Bronfenbrenner’s ecological systems theory (EST) [18, 19], we focused on proximal ecological determinants, as the broader exosystem and macrosystem were constant in this single‐site sample. Therefore, we selected predictors representing two salient ecological layers: the individual and microsystem levels. This study aims to identify distinct time management profiles among nursing interns and examine associated factors within this theoretical framework. The findings will provide empirical evidence for nursing managers and educators to implement stratified management and precise interventions.

## 2. Methods

### 2.1. Theoretical Framework and Variable Selection

Guided by EST, which posits that behavioral outcomes are concurrently associated with individual and environmental characteristics, we constructed a conceptual framework to examine time management among nursing interns. Rather than testing the full ecological model, this study selectively drew on its individual‐ and microsystem‐level constructs. The transition from school to clinical internship represents a critical shift in nursing students’ lives, during which they typically experience reduced family contact and consequent changes in family functioning. Nevertheless, family functioning remains a proximal microsystem that continuously shapes interns’ adaptation; accordingly, it was measured as perceived family functioning during the internship period to capture its post‐transition status [20]. At the individual level, mobile phone addiction [21] and general self‐efficacy [22] were included as key factors. Given the observed heterogeneity in time management, LPA was adopted to identify distinct time management profiles, with mobile phone addiction, family functioning, and general self‐efficacy examined as correlates of profile membership.

### 2.2. Participants

An online survey was conducted from May to July 2025 among nursing interns at a provincial tertiary hospital in Anhui Province. Participants were recruited via convenience sampling from eight nursing colleges whose students were completing clinical internships at this hospital. The sample included four undergraduate/graduate institutions (*n* = 152) and four vocational colleges (*n* = 139). The specific sample sizes for each institution were 12, 80, 37, 23, 13, 60, 40, and 26, respectively. Inclusion criteria were (1) being a full‐time clinical nursing intern and (2) providing written informed consent. Exclusion criteria were (1) absence from the internship exceeding 1 month and (2) less than 2 weeks of clinical experience (e.g., observers). Determining the appropriate sample size for LPA is methodologically complex. As statistical power depends on multiple factors, including the number of latent profiles, the degree of class separation, and the relative proportions of each class, consequently, there is no universally accepted formula for sample size estimation in LPA [23, 24]. However, methodological guidelines suggest that LPA typically requires 50–100 cases per expected profile to ensure reliable classification. Based on the anticipated three‐profile solution, a sample size range of 150–300 participants was deemed necessary. Furthermore, simulation studies have demonstrated that LPA can achieve stable parameter estimation with sample sizes of approximately 200–500 participants. The current study’s sample size (*n* = 291) falls within these recommended empirical ranges, ensuring sufficient power for the exploratory identification of time management profiles.

### 2.3. Measurement

#### 2.3.1. Demographic Information

Demographic data were collected using a self‐designed questionnaire assessing age, gender, educational level, internship duration, intention for further education, family residence, only‐child status, and single‐parent family status.

#### 2.3.2. Adolescence Time Management Disposition Scale (ATMD)

The ATMD, developed by Huang and Zhang [25], was used to assess time management. This 44‐item instrument comprises three primary dimensions (sense of time value, sense of time control, and sense of time efficacy) and nine sub‐dimensions (social orientation, personal orientation, goal setting, plan implementation, priority ranking, time allocation, monitoring feedback, time management efficacy, and practical management behavior efficacy). Items are rated on a 5‐point Likert scale (1 = *completely disagree* to 5 = *completely agree*), with total scores ranging from 44 to 220. Higher scores indicate a more effective time management disposition. Items 9, 17, 27, 30, and 41 are reverse‐scored. The scale has demonstrated sound psychometric properties, with internal consistency (*Cronbach’s α*) for its dimensions ranging from 0.61 to 0.81 and test–retest reliability from 0.71 to 0.85. Previous studies have supported its content and construct validity. In the present study, *Cronbach’s α* for the total scale was 0.927, indicating excellent internal consistency.

#### 2.3.3. Mobile Phone Addiction Index (MPAI)

The MPAI, originally developed by Leung [26] and adapted by Huang et al. [27], is a 17‐item scale comprising four dimensions: loss of control, withdrawal, anxiety and feeling lost, and inefficiency. Items are scored on a 5‐point Likert scale (1 = never to 5 = always). Total scores range from 17 to 85, with higher scores indicating a greater tendency for mobile phone addiction. The scale has demonstrated good internal consistency reliability in previous research (*Cronbach’s α* = 0.91). In the present study, *Cronbach’s α* for the total scale was 0.971, indicating excellent internal consistency.

#### 2.3.4. Family APGAR Index (APGAR)

The APGAR, developed by Smilkstein [28], assesses an individual’s satisfaction with family function across five dimensions: adaptability, partnership, growth, affection, and resolve. This 5‐item scale uses a 3‐point scoring system (0–2), with total scores ranging from 0 to 10. The Chinese version of the scale [29] has demonstrated good test–retest reliability (*r* = 0.80–0.83). In the present study, *Cronbach’s α* for the scale was 0.623, indicating acceptable internal consistency.

#### 2.3.5. General Self‐Efficacy Scale (GSES)

The GSES, developed by Schwarzer and colleagues and validated in a Chinese version by Caikang Wang and Yong [30], was used to assess self‐efficacy. Items are rated on a 4‐point Likert scale (1 = not at all true to 4 = exactly true). Total scores range from 10 to 40, with higher total scores indicating greater self‐efficacy. The scale has previously demonstrated good internal consistency reliability (*Cronbach’s α* = 0.87). In the present study, *Cronbach’s α* for the scale was 0.891, indicating excellent internal consistency.

### 2.4. Data Collection

Data were collected through an electronic questionnaire hosted on the “Wenjuanxing” platform. Nursing managers distributed the survey link and quick response (QR) code to interns via university WeChat groups. Managers explained the study’s purpose and encouraged clinical instructors to facilitate intern participation, while head nurses provided reminders as needed to maximize response rates. Before accessing the survey, participants viewed an informed consent page detailing the study objectives; clicking “Agree” granted access to the questionnaire. To prevent duplicate submissions, the platform was configured to allow only one response per IP address. Two researchers independently screened all submissions for validity. Responses were excluded if completion time was abnormally short (< 120 s) or exhibited patterned responses (e.g., straight‐lining). The final verified dataset was exported to Microsoft Excel for accuracy checks before statistical analysis.

### 2.5. Statistical Analysis

LPA was conducted using R Version 4.3.2 with the tidyLPA package to identify latent profiles based on Time Management Disposition scores. Models were estimated iteratively, starting with a one‐profile solution and incrementally adding profiles. Model selection was guided by the requirement that the smallest profile comprise ≥ 5% of the total sample and that the model remain theoretically interpretable. Model fit was evaluated using Akaike’s information criterion (AIC), Bayesian information criterion (BIC), and sample‐size‐adjusted BIC (aBIC), with lower values indicating better fit. Classification quality was assessed using entropy, with values closer to 1 indicating more precise classification. The Lo–Mendell–Rubin adjusted likelihood ratio test (LMR‐LRT) and bootstrapped likelihood ratio test (BLRT) were also used; a significant *p* value (< 0.05) indicated that a k‐profile model fit better than a *k* − 1‐profile model. The final model was selected based on statistical indices, theoretical interpretability, and profile sizes. All other analyses were performed using SPSS 25.0. Continuous variables were presented as mean ± standard deviation (SD) or median (interquartile range [IQR]) depending on their distribution, while categorical variables were summarized as frequencies and percentages. Differences across latent profiles were compared using chi‐square tests for categorical variables and nonparametric tests (Mann–Whitney *U* or Kruskal–Wallis tests) for continuous variables (MPAI, Family APGAR, and GSES scores). Multinomial logistic regression was used to identify factors associated with profile membership. Independent variables that showed statistical significance (*p* < 0.05) in the univariate analyses were subsequently included in the multivariable model. Harman’s single‐factor test [31] was used to examine potential common method bias. All items were subjected to an unrotated exploratory factor analysis. Common method bias was considered problematic if the first factor accounted for the majority of the total variance (e.g., > 40%–50%). In this study, the first factor explained 30.88% of the total variance, which was below the threshold, indicating the absence of severe common method bias.

### 2.6. Quality Control

To ensure data quality, strict quality control measures were implemented throughout the study. First, a pilot study involving 10 nursing interns was conducted to assess the feasibility and clarity of the questionnaire. Second, as described in the data collection procedures, settings restricting submissions to one per IP address and device were enabled to minimize duplicate entries. Third, settings restricting submissions to one per IP address and device were enabled to minimize duplicate entries. Finally, data verification and cleaning were performed independently by two researchers, who excluded invalid responses based on abnormal response times, logical inconsistencies, or straight‐lining patterns.

### 2.7. Ethics Approval

The study protocol was conducted in accordance with the ethical principles of the Declaration of Helsinki (2013 revision) and was approved by the Institutional Review Board (IRB) of the First Affiliated Hospital of Anhui Medical University (Approval No.: PJ2025‐06‐44). Prior to enrollment, all participants were informed about the study’s purpose and assured of anonymity and confidentiality, after which they provided electronic informed consent.

## 3. Results

### 3.1. Demographic Characteristics

A total of 306 questionnaires were collected. After excluding 15 invalid responses, the final sample comprised 291 participants, including 246 (84.5%) females and 45 (15.5%) males, with a mean age of 20.55 years (SD = 1.23). In terms of educational background, 140 (48.1%) were undergraduates, 126 (43.3%) were junior college students, 13 (4.5%) were secondary vocational students, and 12 (4.1%) were postgraduates. Regarding internship duration, 158 (54.3%) were in their first month, 109 (37.5%) had completed 1–2 months, 18 (6.2%) had completed 2‐3 months, and 6 (2.1%) had completed more than 3 months. The median score for overall time management disposition was 173.00 (IQR: 158.00–187.00). The median (IQR) scores for the three primary dimensions (sense of time value, sense of time control, and sense of time efficacy) were 40.00 (36.00–44.00), 94.00 (85.00–101.00), and 39.00 (36.00–42.00), respectively. Table 1 summarizes the demographic characteristics of the participants.

**TABLE 1:** Demographic characteristics of nursing interns (*n* = 291).

Variable		*n* (%)/mean ± SD
Age		20.55 ± 1.23

Gender	Male	45 (15.5)
	Female	246 (84.5)

Educational level	Associate degree and below	139 (47.8)
	Bachelor’s degree and above	152 (52.2)

Intention for further education	No	89 (30.6)
	Yes	202 (69.4)

Family residence	Rural area	193 (66.3)
	Urban area	98 (33.7)

Only‐child status	Yes	40 (13.7)
	No	251 (86.3)

Single‐parent family status	Yes	32 (11.0)
	No	259 (89.0)

Internship duration	First month	158 (54.3)
	1‐2 months	109 (37.5)
	2‐3 months	18 (6.2)
	> 3 months	6 (2.1)

### 3.2. LPA of ATMD

To identify the optimal latent profile model, solutions with 1–6 classes were estimated (Table 2). Although the AIC, BIC, and aBIC values continued to decrease up to the 6‐class solution, the magnitude of improvement attenuated significantly after the 3‐class solution [32]. While the LMR and BLRT tests remained significant for higher order models, the 3‐class solution demonstrated superior classification quality, yielding the highest entropy (0.965) compared to more complex models (e.g., 0.948 for the 4‐class solution). Moreover, analysis of the 4‐, 5‐, and 6‐class solutions revealed class fragmentation, introducing marginal subgroups (e.g., a 7.5% subgroup in the 4‐class model). In the context of nursing internship education, such small subsets may represent statistical outliers rather than distinct, stable subpopulations with practical implications for intervention. Accordingly, the 3‐class model was retained based on parsimony and clinical utility. The final model included three profiles: Class 1 (*n* = 48, 16.5%), Class 2 (*n* = 175, 60.1%), and Class 3 (*n* = 68, 23.4%), as illustrated in Figure 1.

**TABLE 2:** Fit indices for latent profile models of time management disposition (*n* = 291).

Model	AIC	BIC	aBIC	Entropy	*p*		Class probabilities
					LMR	BLRT	
1	13326.39	13392.51	13335.43	—	—	—	—
2	12155.66	12258.51	12169.72	0.903	*p* < 0.001	0.01	0.605/0.395
3	11134.30	11273.89	11153.38	0.965	*p* < 0.001	0.01	0.601/0.165/0.234
4	10825.42	11001.74	10849.52	0.948	*p* < 0.001	0.01	0.267/0.075/0.456/0.201
5	10680.84	10893.90	10709.97	0.945	*p* < 0.001	0.01	0.433/0.247/0.075/0.127/0.118
6	10634.90	10884.69	10669.05	0.949	*p* < 0.001	0.01	0.436/0.235/0.074/0.084/0.057/0.114

*Note:* aBIC = sample‐size‐adjusted BIC; Entropy = index of classification accuracy; LMRT = Lo–Mendell–Rubin adjusted likelihood ratio test; *p* values for LMRT and BLRT indicate whether a *k*‐profile model fits significantly better than a *k* − 1 profile model. Lower AIC, BIC, and aBIC values indicate a better fit; Entropy values 0.80 indicate good classification accuracy.

Abbreviations: AIC, Akaike information criterion; BIC, Bayesian information criterion; BLRT, bootstrap likelihood ratio test.

**FIGURE 1:**
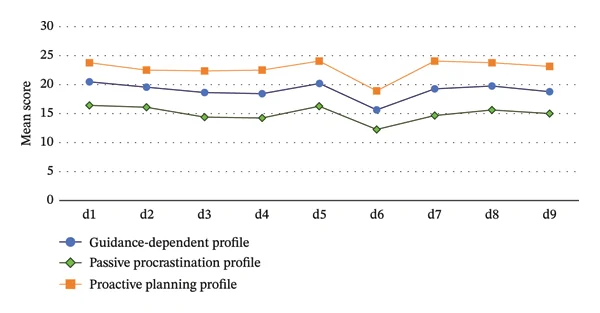
Latent profiles of time management disposition in nursing interns (*n* = 291).

Class 1 (*n* = 48): Passive Procrastination Profile. This profile exhibited relatively low scores on key dimensions such as time allocation (*M* = 12.20) and goal setting (*M* = 14.42). These scores suggest suboptimal time management, characterized by procrastination, such as failing to complete tasks on time and lacking execution motivation.

Class 2 (*n* = 175): Guidance‐Dependent Profile. This profile scored moderately on external coordination dimensions, such as social orientation (*M* = 20.40) and personal orientation (*M* = 19.47). However, it demonstrated lower scores on self‐regulation dimensions, such as monitoring feedback and time allocation (e.g., time allocation *M* = 15.55, notably lower than the 18.94 observed in the Proactive Planning Profile). This pattern suggests a reliance on external guidance for reminders and scheduling.

Class 3 (*n* = 68): Proactive Planning Profile. This profile scored highest on active regulation dimensions, such as goal setting, plan implementation, and priority ranking (e.g., goal setting *M* = 22.41; priority ranking *M* = 24.02). This reflects strong self‐regulatory abilities, characterized by proactive goal setting, plan formulation, and effective execution.

### 3.3. Comparison of Characteristics Across Latent Classes

Significant differences in demographic and psychological characteristics were observed among the three latent profiles (Table 3). Post‐hoc pairwise comparisons indicated that the Proactive Planning Profile exhibited a higher educational level than the Passive Procrastination and Guidance‐Dependent profiles (both *p* < 0.05). The Guidance‐Dependent profile demonstrated the lowest intention to pursue further education among the three groups. Regarding psychological and environmental factors, the Proactive Planning Profile showed higher scores on the Family APGAR and GSES, as well as lower scores on the MPAI, compared to the other two profiles.

**TABLE 3:** Comparison of demographic and psychological characteristics across latent profiles (*n* = 291).

Variable	Class 1 (*n* = 48)	Class 2 (*n* = 175)	Class 3 (*n* = 68)	*χ* ^2^/*H*	*p*
Gender				0.083	0.959
male	8	27	10		
female	40	148	58		
Education level				23.555	*p* < 0.001
Associate degree and below	26	98	15		
Bachelor’s degree and above	22	77	53		
Intention for further education				21.029	*p* < 0.001
No	9	71	9		
Yes	39	104	59		
Family residence				0.390	0.823
Rural area	30	117	46		
Urban area	18	58	22		
Only‐child status				0.121	0.941
No	42	150	59		
Yes	6	25	9		
Single‐parent family status				2.657	0.265
No	43	152	64		
Yes	5	23	4		
MPAI	52.00 (48.00, 55.00)	50.00 (45.00, 57.00)	45.50 (39.00, 50.00)	26.413	*p* < 0.001
APGAR	7.00 (6.00, 8.00)	7.00 (6.00, 8.00)	9.00 (8.25, 10.00)	82.391	*p* < 0.001
GSES	27.00 (23.00, 28.75)	27.00 (24.00, 30.00)	32.00 (30.00, 36.00)	66.906	*p* < 0.001

*Note:* Continuous variables are presented as median (interquartile range). APGAR = Family APGAR Index; *χ* ^2^ = chi‐square test; *H* = Kruskal–Wallis test.

Abbreviations: GSES, General Self‐Efficacy Scale; MPAI, Mobile Phone Addiction Index.

### 3.4. Factors Influencing ATMD Profiles

95% confidence intervals (CIs) and regression coefficients are presented in Table 4. Prior to model selection, the proportional odds assumption for ordinal logistic regression was tested and violated (parallel lines test: *χ* ^2^ = 34.493, df = 2 *p* < 0.05); therefore, multinomial logistic regression was performed to identify factors associated with latent profile membership. The three latent profiles identified via LPA were entered as the dependent variable (categorical), whereas the APGAR, GSES, and MPAI scores were entered as independent variables. Variables significant in univariate analyses (*p* < 0.05) were retained in the multivariable model. Categorical variables, specifically educational level (associate degree and below = 0; bachelor’s degree and above = 1) and intention for further education (No = 0; Yes = 1), were dummy coded, with “bachelor’s degree and above” and “Yes” set as the reference categories. Continuous predictors, including the APGAR, GSES, and MPAI scores, were entered directly into the model. The Proactive Planning Profile was set as the reference category. Lower APGAR and GSES scores were significantly associated with membership in both the Passive Procrastination and Guidance‐Dependent Profiles, relative to the Proactive Planning Profile. Additionally, lacking an intention to pursue further education was uniquely associated with membership in the Guidance‐Dependent profile. Adjusted odds ratios with 95% CIs and regression coefficients are presented in Table 4.

**TABLE 4:** Multinomial logistic regression of factors associated with latent profile membership (*n* = 291).

Model	*B*	SE	Wald *χ* ^2^	*p*	OR	95% CI
*Class 1 vs. Class 3*						
Intercept	15.560	3.18	23.948	—	—	—
Educational level	−0.132	0.542	0.060	0.807	0.876	0.303–2.532
Intention for further education	0.320	0.598	0.256	0.613	1.353	0.419–4.371
APGAR	−1.160	0.216	28.728	*p* < 0.001	0.313	0.205–0.479
GSES	−0.252	0.056	20.255	*p* < 0.001	0.777	0.696–0.867
MPAI	0.015	0.033	0.212	0.645	1.015	0.952–1.082

*Class 2 vs. Class 3*						
Intercept	13.678	2.744	24.842	—	—	—
Educational level	0.088	0.460	0.036	0.849	1.092	0.443–2.689
Intention for further education	1.358	0.472	8.279	0.004	3.888	1.542–9.805
APGAR	−0.958	0.189	25.575	*p* < 0.001	0.384	0.265–0.556
GSES	−0.197	0.046	18.352	*p* < 0.001	0.822	0.751–0.899
MPAI	0.012	0.027	0.207	0.649	1.012	0.960–1.067

*Note:* The reference category for the dependent variable was Class 3 (Proactive Planning profile). For independent variables, the reference categories were “bachelor’s degree and above” for educational level and “Yes” for intention for further education. *B* = unstandardized coefficient.

Abbreviations: CI, confidence interval; OR, odds ratio; SE, standard error.

## 4. Discussion

### 4.1. Heterogeneity of Time Management Disposition Among Nursing Interns

This cross‐sectional study identified three distinct time management profiles among nursing interns: the Passive Procrastination Profile (16.5%), the Guidance‐Dependent Profile (60.1%), and the Proactive Planning Profile (23.4%). These findings highlight the heterogeneity of time management disposition within this population. The observed differences from the findings of studies involving nursing managers [9, 14] may be attributed to variations in professional maturity and measurement tools. Importantly, these distinct profiles suggest that time management is not a unitary construct, supporting the implementation of stratified management approaches in clinical education.

The majority of interns (60.1%) were classified into the Guidance‐Dependent Profile. This group exhibited a relative strength in orientation dimensions but weaknesses in time allocation, suggesting a potential regulation gap between motivation and execution [33]. Their reliance on external prompts appears to serve as a compensatory mechanism [34–36]. This phenomenon may be partially shaped by the Chinese educational context, where exam‐oriented systems and hierarchical clinical training prioritize compliance and execution over fostering independent decision‐making and proactive planning. For this large transitional subgroup, nursing managers could implement scaffolding strategies such as providing visual management tools [37] initially and gradually encouraging independent planning to facilitate the shift from external reliance to internal autonomy. The Passive Procrastination Profile, characterized by low scores across self‐regulation dimensions, suggests that interns in this group may struggle with the forethought phase of task management. Consequently, they require support beyond basic skill training. Targeted interventions focusing on psychological capital and micro‐goal setting may be beneficial. Creating a psychologically safe environment that allows for trial and error could help mitigate the avoidance behaviors often associated with procrastination [16, 38, 39]. In contrast, the Proactive Planning Profile demonstrated high self‐monitoring capabilities, indicating that these individuals possess mature regulatory skills. Utilizing these individuals as peer mentors [40] might be a viable strategy to foster a supportive learning ecosystem. This approach not only aids lower performing interns but also refines the leadership competencies of high‐potential individuals [41, 42]. Given the cross‐sectional design, causal relationships cannot be confirmed. Future longitudinal or intervention studies are warranted to test the effectiveness of these stratified strategies.

### 4.2. Influencing Factors of Time Management Disposition Profiles Among Nursing Interns

#### 4.2.1. Intention for Further Education

Notably, the Passive Procrastination and Proactive Planning Profiles did not differ significantly in their intention to pursue further education; however, the former exhibited lower time management efficiency, indicating a pronounced intention–behavior gap. This gap may partly reflect individual differences in internal and familial resources for translating intentions into action rather than a lack of motivation. Nursing interns with lower self‐efficacy may doubt their capacity to execute study plans amid competing demands, whereas supportive family environments may provide the emotional stability and external structure that facilitate such self‐regulatory efforts. Alternative explanations include defensive procrastination [43] and unmeasured demands of the clinical learning environment [44], although the latter remains speculative because clinical microsystem variables were not measured in the present study. To bridge this gap, nursing managers could provide behavioral scaffolds such as checklists [45] and visual flowcharts as external regulatory aids that help interns translate professional aspirations into actual performance. In contrast, the Guidance‐Dependent Profile exhibited significantly lower motivation for further education alongside poorer time management. This motivational difference remained significant after adjusting for family functioning, self‐efficacy, and mobile phone addiction, indicating an independent motivational disengagement rather than an artifact of weaker personal and familial resources. This pattern may be related to learned helplessness [46]; a perceived lack of control over clinical outcomes may undermine long‐term goal engagement. However, this interpretation remains speculative and requires direct measurement of clinical microsystem variables for verification. Accordingly, interventions for this profile could prioritize psychological empowerment and motivational enhancement [47], with mentorship and structured career planning helping these interns build competence and shift from passive adaptation to active participation.

#### 4.2.2. General Self‐Efficacy

Our findings indicated that high self‐efficacy was significantly associated with membership in the Proactive Planning Profile, distinguishing it from both of the less adaptive profiles. This difference may partly reflect differences in cognitive appraisal processes. The clinical learning environment is widely regarded as dynamic and unpredictable, and interns frequently confront unplanned events, such as sudden changes in patients’ conditions [48]. In such settings, interns with high self‐efficacy tend to appraise these demands as challenges rather than threats [49]. This adaptive appraisal may preserve self‐regulatory resources, enabling flexible adjustment of task priorities rather than cognitive paralysis [50]. These appraisal differences may partly explain the observed disparities in time management across profiles. Moreover, the present findings showed that high self‐efficacy was associated with proactive goal setting, while the literature suggests that successful time management experiences may, in turn, enhance self‐efficacy [51]. This reciprocal association likely constitutes a core psychological characteristic distinguishing the Proactive Planning Profile; however, the underlying pathway cannot be established given the cross‐sectional design of the present study. For interns with low self‐efficacy, clinical preceptors should shift their focus from teaching techniques to rebuilding confidence. Specifically, preceptors can facilitate mastery experiences by decomposing complex tasks into manageable steps and promote vicarious learning by pairing interns with Proactive Planning Profile peers as mentors. Additionally, strategy‐oriented feedback [52] and structured tools, such as checklists, can serve as external scaffolds that reduce cognitive load and help interns develop autonomous time management habits.

#### 4.2.3. Family APGAR Index

In the context of Chinese culture, the family functions as a microsystem characterized by shared responsibilities [53], in which family‐provided support is closely linked to the accumulation of individual psychological resources. Empirical evidence indicates that favorable family functioning is associated with fewer adaptive barriers in clinical practice and better allocation of cognitive resources, whereas insufficient family support is linked to compounded clinical stress and emotional depletion, increasing the likelihood of passive procrastination [54]. The relatively low *Cronbach’s α* for the Family APGAR Index in this study may reflect interns’ critical transition from the family to the professional microsystem. During the early internship period, dimensions of family functioning may evolve asynchronously: material and emotional support typically remain high, whereas tensions may emerge in the dimensions of adaptation and growth as interns strive for independence. This dimensional heterogeneity may partly explain the reduced internal consistency, reflecting the complex renegotiation of support and control during this clinical adjustment phase [55]. Despite potential attenuation from measurement error, the observed significant association underscores the robustness of the relationship between family functioning and time management. Notably, family support may exhibit nonlinear effects during early clinical transitions. Although it serves as a buffer, excessive family intervention may be associated with difficulties in developing autonomous self‐regulation. If family support fails to transition from direct involvement to fostering independence, interns may experience challenges in self‐regulation [56], which may correlate with their time management behaviors. Future research should explore the dynamic evolution of family functioning, specifically examining the threshold at which family support shifts from a protective factor to a restrictive one.

A notable finding is that although the MPAI score was significantly associated with time management profile membership in univariate analyses, this association became nonsignificant in the multivariable multinomial logistic regression after adjustment for family functioning and general self‐efficacy. This finding is inconsistent with the results reported by El‐Sayed et al. [57]. From a statistical perspective, this attenuation suggests that the bivariate relationship between mobile phone addiction and time management may be largely explained by shared variance with family functioning and self‐efficacy. This interpretation is consistent with existing literature showing that mobile phone addiction is conceptually intertwined with low self‐efficacy and inadequate family functioning [58]. It is therefore plausible that mobile phone addiction co‐occurs with these underlying psychological and familial factors rather than being independently associated with time management profile membership. This pattern also aligns with the perspective that mobile phone addiction functions as a maladaptive coping strategy [59, 60], co‐occurring with rather than directly causing poor time management. Nevertheless, several caveats should be acknowledged. First, the cross‐sectional design precludes causal inference regarding the direction of these relationships. Second, the mediating pathways implied by the coping‐strategy account were not formally tested. Accordingly, the above interpretation remains speculative and requires empirical validation through longitudinal designs.

### 4.3. Implications to Nursing Practice and Research

Interpreted through the framework of EST, the findings of this study support profile‐specific nursing management strategies. For the Guidance‐Dependent Profile, interventions should target the individual system through incremental task assignments designed to foster self‐efficacy, complemented by structured clinical scaffolding within the microsystem to facilitate autonomy. At the individual level, interns in the Passive Procrastination Profile, unlike those in the Guidance‐Dependent Profile, retain a positive intention to pursue further education, suggesting that this group requires targeted behavioral guidance rather than motivational enhancement. For this profile, nurse managers should establish early warning mechanisms to identify interns with declining time management performance and cultivate psychologically safe environments that reduce the fear of disclosure and help‐seeking. At the family microsystem level, interventions could encourage families to shift from providing basic life care to offering growth‐oriented support, such as facilitating interns’ scheduling autonomy and emotional encouragement. In contrast, for the Proactive Planning Profile, strategies should prioritize sustaining existing adaptive behaviors and leveraging these interns’ strengths within the clinical microsystem. Engaging these interns as peer mentors can create a synergistic environment in which their robust time management skills facilitate the development of peers’ self‐regulation. Simultaneously, providing advanced clinical learning opportunities at the individual level can help maintain their motivation for professional development and prevent early career stagnation.

Finally, although mobile phone addiction was not an independent correlate of profile membership in the multivariable model, cultivating digital self‐regulation skills remains essential to help interns manage attention within demanding, multitasking clinical environments. Ultimately, this ecosystem‐based approach enables nurse administrators to move beyond homogeneous, one‐size‐fits‐all teaching models toward stratified, precision management strategies tailored to interns’ ecological contexts.

## 5. Limitations

This study has several limitations. First, the cross‐sectional study design precludes causal inferences and prohibits evaluations of the temporal stability of the identified latent profiles. As such, the extracted profiles may reflect temporary transitional states instead of stable, enduring personal traits. Second, the exclusive use of self‐reported data may introduce response bias, potentially compromising the validity of the study findings. Furthermore, the ATMD scale, developed in 2001, may not fully capture modern time management challenges, including digital distractions and frequent clinical multitasking, which may limit its ecological validity in current clinical practice settings. Third, the single‐center design and moderately limited sample size restrict the generalizability of the results. Although participants were recruited from eight nursing colleges with internships at the same tertiary hospital, the small number of college clusters prevented the modeling of school‐level clustering effects (e.g., differences in curriculum design and training systems). This omission may have resulted in underestimated standard errors and inflated Type I error rates. Fourth, although we adopted the BLRT and followed multicriteria guidelines for latent profile model selection, supplementary bootstrap‐assisted class enumeration failed to achieve model convergence. This issue likely stemmed from high model dimensionality (nine indicators) relative to the limited sample size of individual subgroups. While the three‐profile solution is theoretically reasonable and interpretable, statistical stability remains uncertain for the optimal latent profile classification, especially for the smallest subgroup. Finally, several potential confounding and influential factors were not measured in this study, including individual‐level variables (e.g., student leadership experience) and clinical microsystem variables. Future research should adopt large‐scale, multicenter sampling, longitudinal study designs, and multilevel modeling approaches to address the above limitations.

## 6. Conclusion

This study employed LPA to identify distinct time management profiles among nursing interns, revealing significant heterogeneity across three classes: the Passive Procrastination, Guidance‐Dependent, and Proactive Planning Profiles. In this cross‐sectional sample, interns reporting a strong intention to pursue further education, high general self‐efficacy, and favorable family functioning were more likely to be classified as Proactive Planners. Based on these findings, we recommend that nurse managers and clinical preceptors implement tailored, profile‐specific time management interventions. These strategies should provide structured support to navigate competing demands within the clinical microsystem while simultaneously addressing individual psychological needs. By integrating interventions at both the clinical and individual levels rather than relying on a uniform approach, clinical educators can optimize the support environment, ultimately facilitating interns’ clinical adaptation and professional development.

## Funding

The authors received no specific funding for this work.

## Conflicts of Interest

The authors declare no conflicts of interest.

## Data Availability

The data that support the findings of this study are available from the corresponding author upon reasonable request.
